# Antitubercular, Cytotoxicity, and Computational Target Validation of Dihydroquinazolinone Derivatives

**DOI:** 10.3390/antibiotics11070831

**Published:** 2022-06-21

**Authors:** Katharigatta N. Venugopala, Nizar A. Al-Shar’i, Lina A. Dahabiyeh, Wafa Hourani, Pran Kishore Deb, Melendhran Pillay, Bashaer Abu-Irmaileh, Yasser Bustanji, Sandeep Chandrashekharappa, Christophe Tratrat, Mahesh Attimarad, Anroop B. Nair, Nagaraja Sreeharsha, Pottathil Shinu, Michelyne Haroun, Mahmoud Kandeel, Abdulmalek Ahmed Balgoname, Rashmi Venugopala, Mohamed A. Morsy

**Affiliations:** 1 Department of Pharmaceutical Sciences, College of Clinical Pharmacy, King Faisal University, Al-Ahsa 31982, Saudi Arabia; ctratrat@kfu.edu.sa (C.T.); mattimarad@kfu.edu.sa (M.A.); anair@kfu.edu.sa (A.B.N.); sharsha@kfu.edu.sa (N.S.); mharoun@kfu.edu.sa (M.H.); abalgoname@kfu.edu.sa (A.A.B.); momorsy@kfu.edu.sa (M.A.M.); 2 Department of Biotechnology and Food Science, Faculty of Applied Sciences, Durban University of Technology, Durban 4000, South Africa; 3 Department of Medicinal Chemistry and Pharmacognosy, Faculty of Pharmacy, Jordan University of Science and Technology, P.O. Box 3030, Irbid 22110, Jordan; nashari@just.edu.jo; 4 Department of Pharmaceutical Sciences, School of Pharmacy, The University of Jordan, Amman 11942, Jordan; l.dahabiyeh@ju.edu.jo; 5 Department of Pharmaceutical Sciences, Faculty of Pharmacy, Philadelphia University, Amman 19392, Jordan; wafa.hourani@hotmail.com; 6 Department of Microbiology, National Health Laboratory Services, KZN Academic Complex, Inkosi Albert Luthuli Central Hospital, Durban 4001, South Africa; melendhra.pillay@nhls.ac.za; 7 Hamdi Mango Center for Scientific Research, The University of Jordan, Amman 11942, Jordan; abuirmailehbashaer@yahoo.com (B.A.-I.); bustanjiyasser@gmail.com (Y.B.); 8 Department of Basic Medical Sciences, College of Medicine, University of Sharjah, Sharjah 27272, United Arab Emirates; 9 Department of Medicinal Chemistry, National Institute of Pharmaceutical Education and Research (NIPER-R) Raebareli, Lucknow 226002, India; c.sandeep@niperraebareli.edu.in; 10 Department of Pharmaceutics, Vidya Siri College of Pharmacy, Off Sarjapura Road, Bangalore 560035, India; 11 Department of Biomedical Sciences, College of Clinical Pharmacy, King Faisal University, Al-Ahsa 31982, Saudi Arabia; spottathail@kfu.edu.sa; 12 Department of Biomedical Sciences, College of Veterinary Medicine, King Faisal University, Al-Ahsa 31982, Saudi Arabia; mkandeel@kfu.edu.sa; 13 Department of Pharmacology, Faculty of Veterinary Medicine, Kafrelsheikh University, Kafrelsheikh 33516, Egypt; 14 Department of Public Health Medicine, Howard College Campus, University of KwaZulu-Natal, Durban 4001, South Africa; rashmivenugopala@gmail.com; 15 Department of Pharmacology, Faculty of Medicine, Minia University, El-Minia 61511, Egypt

**Keywords:** dihydroquinazolin-4(1*H*)-ones, anti-TB activity, MTT assay, molecular docking studies, molecular dynamic simulations studies

## Abstract

A series of 2,3-dihydroquinazolin-4(1*H*)-one derivatives (**3a**–**3m**) was screened for in vitro whole-cell antitubercular activity against the tubercular strain H37Rv and multidrug-resistant (MDR) *Mycobacterium tuberculosis* (MTB) strains. Compounds **3l** and **3m** with di-substituted aryl moiety (halogens) attached to the 2-position of the scaffold showed a minimum inhibitory concentration (MIC) of 2 µg/mL against the MTB strain H37Rv. Compound **3k** with an imidazole ring at the 2-position of the dihydroquinazolin-4(1*H*)-one also showed significant inhibitory action against both the susceptible strain H37Rv and MDR strains with MIC values of 4 and 16 µg/mL, respectively. The computational results revealed the mycobacterial pyridoxal-5′-phosphate (PLP)-dependent aminotransferase (BioA) enzyme as the potential target for the tested compounds. In vitro, ADMET calculations and cytotoxicity studies against the normal human dermal fibroblast cells indicated the safety and tolerability of the test compounds **3k**–**3m**. Thus, compounds **3k**–**3m** warrant further optimization to develop novel BioA inhibitors for the treatment of drug-sensitive H37Rv and drug-resistant MTB.

## 1. Introduction

Tuberculosis (TB) is one of the top 10 leading causes of mortality worldwide and is the leading cause of death from infectious disease among adults [1]. It is a communicable disease caused by the bacterium *Mycobacterium tuberculosis* (MTB) that primarily affects the lungs, resulting in pulmonary TB [2]. TB can also infect other sites of the body, causing extrapulmonary TB [3,4].

To date, no major changes in the treatment of TB have been made, and the current standard treatment still involves a combination of four antibiotics (isoniazid, rifampin, pyrazinamide, and ethambutol) given for two months followed by isoniazid and rifampicin for an additional four months [2]. This anti-TB regimen has been successful in the treatment of MTB H37Rv. However, the emergence of multidrug-resistant TB (MDR-TB) and extensively drug-resistant TB (XDR-TB), as well as HIV/TB co-infection cases, have made TB control more difficult [5,6]. Moreover, treating resistant TB can take up to 24 months and might be associated with side effects and a low chance of cure. This, in turn, can lead to poor patient compliance, which can also contribute to the development of resistance [7].

**Figure 1:**
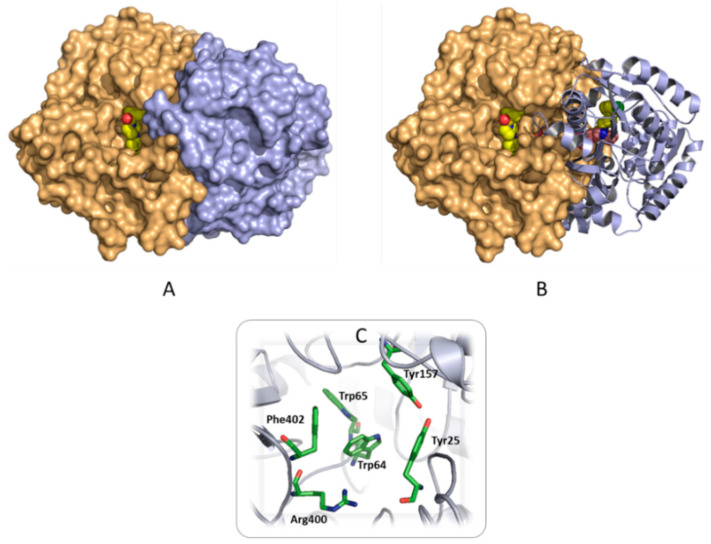
The 3D crystal structure of the *Mycobacterium tuberculosis* BioA enzyme (PDB code 4XJO). (**A**) Surface representation of the homodimer enzyme. Monomer A is colored light brown, and monomer B is colored light blue. The co-crystallized inhibitors are shown in the CPK representation with carbons colored yellow. (**B**) Same view as in A, in which monomer B is shown in cartoon representation to highlight the location of the two active sites. The PLP molecule is shown in the CPK representation with carbons colored pink. (**C**) Detailed representation of the active site highlighting the main binding residues that are represented as sticks with carbons colored green.

Several drug leads from natural products [7,8] and marine organisms [9] and various chemical entities and repurposed drugs, such as linezolid and clofazimine [10,11], have been suggested for the treatment of resistant TB [2]. Some of them have been conditionally approved and recommended by the World Health Organization (WHO), including bedaquiline, delamanid, and pretomanid. However, the majority are still undergoing clinical trials. Despite their favorable anti-TB action, resistance to both bedaquiline and delamanid, due to prolonged duration of therapy, has been reported [12,13]. Conversely, safety concerns were raised about the use of pretomanid, linezolid, and clofazimine for the treatment of TB [2,14,15]. Despite the efforts to discover new anti-TB compounds, current therapies are still facing the development of resistance and poor compliance due to long treatment duration. Therefore, it is evident that there is an urgent need for the development of new potential anti-TB compounds that can act on new molecular targets to overcome drug-resistant MTB strains and to control the wide spread of TB. 2,3-Dihydroquinazolin-4(1*H*)-ones (2,3-DHQs) are fused heterocyclic compounds that exist in natural products such as luotonins A, B, E and F [16,17], tryptanthrin [18], rutaecarpine [19]. 2,3-DHQs possess a broad range of pharmacological properties such as anti-cancer [20,21,22,23,24], antidepressant [25], antidiabetic [26], antifungal [27], antihypertensive [28,29], anti-inflammatory [30,31], antibacterial [32], antioxidant [33], antiviral [34], bronchodilator [35], centrally acting muscle relaxant [36], diuretic [37], sedative and hypnotic [38] agents (Figure 1). Quinazolin derivatives have also been reported as bactericides [39], fungicides [40] and insecticides [41].

We have been interested in identifying potential novel anti-TB compounds from natural sources [42,43], cyclic depsipeptides [44], and synthetic heterocyclic compounds having pharmacophores, such as benzothiazoles, triazolyl 1,2,3,4-tetrahydropyrimidines, dihydropyrimidines, and substituted indolizines as potential antitubercular agents [45,46,47,48,49,50,51,52]. In continuation of our efforts in identifying promising heterocyclic compounds for pharmacological activity [53,54,55,56,57,58,59,60,61,62,63], this study aims to identify potential anti-TB compounds. Herein, we screened a set of our recently reported substituted 2,3-dihydroquinazolin-4(1*H*)-one analogues (except the novel compound **3l**) to assess their potency against different strains of MTB. These compounds were also further investigated to evaluate their cytotoxicity against normal human dermal fibroblast cells. Moreover, we attempted to identify the putative mycobacterial target of those compounds by applying a computational approach.

## 2. Results and Discussion

### 2.1. Antitubercular Activity

The in vitro antitubercular activity of the tested derivatives (**3a**–**3m**) against H37Rv and multi-drug resistant strains of *M. tuberculosis* is presented in Table 1. The in vitro results showed promising antitubercular activities against the susceptible H37Rv *M. tuberculosis* strain with minimum inhibitory concentration (MIC), ranging from 2–128 µg/mL. The most active analogues against the susceptible H37Rv *M. tuberculosis* strain with a MIC value of 2 g/mL were the compounds **3l** and **3m** with a di-substituted aryl moiety (containing electron withdrawing halogens) connected to the 2-position of the quinazoline scaffold. Compound **3k**, which had a MIC of 4 g/mL, likewise demonstrated extremely good inhibitory efficacy against the same strain. The inclusion of an imidazole ring at the 2-position of the quinazoline scaffold resulted in significant inhibitory action against the multi-drug resistant strains of *M. tuberculosis* with a MIC value of 16 µg/mL. None of the remaining analogues had a promising inhibitory activity against the multi-drug-resistant strains of *M. tuberculosis*. Nevertheless, the identified active compounds could be considered as lead molecules that upon further optimization might evolve into more potent drug candidates.

### 2.2. Cytotoxicity Assay

To determine the safety of the test compounds, 2,3-dihydroquinazolin-4(1*H*)-ones (**3a**–**3m**), the MTT (3-[4,5-dimethylthiazol-2-yl]-diphenyltetrazolium bromide) assay was used as tabulated in Table 1. The most active compounds, **3l** and **3m**, were found to be safe and tolerable, at 36% and 31%, respectively, with toxicity against the normal human dermal fibroblast cells at a concentration of 100 µM. (equal to 32 µg/mL for **3I** and 30 µg/mL for **3m**). The previous statement indicates that these two compounds will have IC_50_ values >100 µM (>32 or 30 µg/mL for **3I** and **3m**, respectively) which is higher than their MIC against MTB (2 µg/mL). The same is for **3k**, which showed an MIC value of 4 µg/mL and 16 µg/mL against the H37Rv and MDR strains, respectively. The cytotoxicity of **3k** when tested at 100 µM (=21.4 µg/mL) was 19.2%, which means that its IC_50_ will be much higher than 21.4 µg/mL and higher than the MIC values.

### 2.3. Computational Studies

#### 2.3.1. Searching for a Putative Drug Target

In this study, the biological testing of the anti-TB activity of the title compounds (**3a**–**3m**) was conducted using the whole-cell screening method; therefore, different computational approaches were implemented to identify a possible drug target that could explain the mechanism of action of the tested compounds. The same approach we devised previously [52] was used in this study. In this approach, an exhaustive literature search identified 48 mycobacterial macromolecules that are essential for bacterial survival and persistence that could be potential MTB drug targets (Table S1), of which 21 targets with solved 3D crystal structures were selected for molecular docking studies (Table S2). The 21 potential targets were selected based on their essentiality to mycobacterial growth and survival, and the availability of their solved 3D crystal structures (H37Rv MTB strain). 

The selected protein crystal structures were prepared, solvated and minimized, as previously described [64,65,66], in order to remove any potential artifacts caused by crystal packing. The binding sites were then determined, and all co-crystallized ligands were extracted and redocked into their appropriate binding sites. Ligand redocking was meant to validate the docking procedure prior to docking the tested compounds. A well-defined binding site and an accurate docking algorithm would be able to regenerate the native co-crystallized pose for the redocked ligands, which is usually assessed by calculating the root mean square deviation (RMSD) between the redocked pose and the native ligand. The calculated RMSD measures the accuracy of the docking algorithm; a value less than 2 is adequate, but a value less than 1 would be excellent [67]. In redocking co-crystallized ligands, the CDOCKER docking algorithm was successful in replicating the binding pose of the native ligands with RMSD values ranging from 0.15 to 1.43 Å. Following the validation step, the tested compounds were docked into the binding sites of the 21 selected proteins, and the docked poses were then rescored using 11 different scoring functions. There are four classes of scoring functions, namely forcefield-based, empirical, knowledge-based, and machine-learning-based scoring functions [68]. In this study, in addition to the two CDOCKER forcefield-based scores, which are the -CDOCKER energy (-CDE) and the -CDOCKER interaction energy (-CDIE) scores, another eight empirical scoring functions were used, namely LigScore1 and LigScore2, PLP1 and PLP2, Jain, Ludi1, Ludi2 and Ludi3, in addition to two knowledge-based scoring functions, PMF and PMF04. All of these scoring functions are available in DS, and their output scores are reported as positive values; hence, the higher the score, the higher the binding affinity.

Following the same previous approach, the identification of a putative mycobacterial target for the tested compounds was achieved by calculating the Pearson Correlation Coefficient (*r*) between the computational scores and their respective experimentally determined MIC values [69] (Table 2). A more potent inhibitor would have a low MIC value, which, ideally, would correlate with a high computational score. Therefore, mycobacterial targets that show negative correlations between their MIC values and computational scores were assumed to be putative targets for the tested compounds. 

Table 2 shows that the highest correlation coefficient between the experimental and computational results was obtained with the PMF4 scoring function when the compounds were docked into the active site of the mycobacterial pyridoxal-5′-phosphate (PLP)-dependent aminotransferase (BioA) enzyme (*r* = −0.86). The high negative correlation means that active compounds (low MIC values) are correlated with high docking scores. The value of the correlation coefficient (*r*) determines the strength of association between the two sets of variables. Generally, a value of |*r*| between 0.1–0.3 indicates a small association, 0.3–0.5 is medium, and 0.5–1.0 indicates a large association [66]. Furthermore, this enzyme showed another high correlation with LigScore1 (*r* = −0.63). Accordingly, the tested compounds are predicted to be most likely targeting the BioA enzyme. These findings can be further investigated by other computational methods such as molecular dynamics (MD) simulations. However, the experimental in vitro enzyme assay remains the main conclusive method to validate our computational results. 

#### 2.3.2. Analysis of the Binding Interactions with the Putative Target (BioA)

The identified putative target was the BioA enzyme. This enzyme catalyzes the second step in biotin biosynthesis and is essential for bacterial survival and persistence. Contrary to humans, MTB de novo synthesizes biotin to be utilized by carboxylases in fatty acid metabolism and gluconeogenesis pathways; thereby, BioA is an ideal target for the development of potential antitubercular agents [70].

Structurally, the functional form of the mycobacterial BioA enzyme is a homodimer, in which the monomer structure is composed of two domains, the small domain (amino acid residues 1–60 and 339–437) and the large domain (residues 61–338). Two active sites are located at the interface between the two monomers, 18 Å apart, and are composed of residues Pro24-Ser34, Ser62-Ala67, Arg156-Asp160, His171-Arg181, Gln224-Gly228, and Arg400-Arg403 from one chain, and Met′87-His′97 and Ala′307-Asn′322 from the other chain [70,71,72]. Among these residues, Tyr25, Trp64, Trp65, Tyr157, Arg400, and Phe402 were reported to be of high relevance to ligand binding [72,73] (Figure 1).

**Figure 2:**
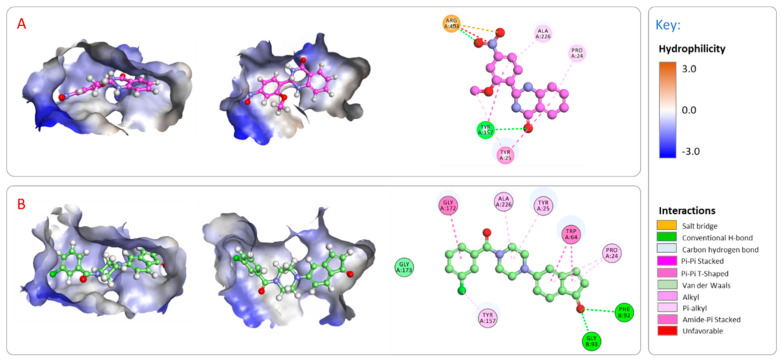
(**A**) The binding mode and binding interactions of compound **3m** within the active site of the BioA enzyme compared to that of the co-crystallized ligand (41O, PDB code 4XJO) in (**B**). The left and middle panels show the binding modes of compound **3m** and 41O. The active site is shown as a hydrophobic surface. The right panel shows the 2D interaction maps of the two compounds. The interacting amino acid residues are shown as disks and are colored according to the type of their intermolecular interactions with the enzyme.

The structural model of the MTB BioA enzyme was downloaded from the protein data bank (PDB code, 4XJO), which corresponds to the BioA enzyme in complex with an inhibitor, 5-[4-(3-chlorobenzoyl)piperazin-1-yl]-1*H*-inden-1-one (41O), and the coenzyme pyridoxal-5′-phosphate (PLP) at a resolution of 1.50 Å; then, it was prepared as detailed in the Methods section. The docking protocol was validated prior to docking the designed compounds via extracting and redocking the co-crystallized ligand (41O). The redocked pose was in perfect agreement with the native co-crystallized ligand pose with a RMSD of 0.15 Å. Then, the designed compounds were docked into the active site of the enzyme. Figure 2 shows the binding mode and binding interactions of compound **3m** within the active site of the BioA enzyme compared to that of the co-crystallized ligand (41O).

As shown in Figure 2, compound **3m** occupies the binding pocket of the active site, and the 2D interaction map shows the involvement of many key binding residues within the active site in intermolecular interactions. Compound **3m** establishes two hydrogen bonds with the amino acid residues Tyr157 and Arg403, a slat bridge with Arg403, along with numerous hydrophobic interactions including pi-pi stacking with Tyr25, pi-pi T-shaped with Tyr157, and pi-alkyl with Pro24 and Tyr25. These interactions suggest that compound **3m** seems to have a favorable binding affinity toward the BioA enzyme. Once the mechanism of action of the tested compounds is confirmed, their detailed binding interactions can be utilized to guide prospective optimization efforts to design more potent and selective drug candidates.

#### 2.3.3. MD Simulations

Compared to molecular docking, MD simulations offer a much higher level of accuracy since the molecular flexibility of the entire simulated complex is fully accounted for. Moreover, MD simulations provide a multitude of dynamical, structural, and energetic information pertaining to the simulated system [74,75]. Therefore, MD was employed herein to further study the binding stability and interactions of the most active compound **3m** with its putative target. The simulated virtual complex was obtained from the top-ranked docked pose of compound **3m** with the BioA enzyme. Moreover, the apo form of the BioA enzyme was also simulated for comparison purposes. The apo form was modeled by deleting the co-crystallized ligands. The generated trajectories were then analyzed based on their thermodynamic properties, RMSD, and root mean square fluctuation (RMSF).

**Figure 3:**
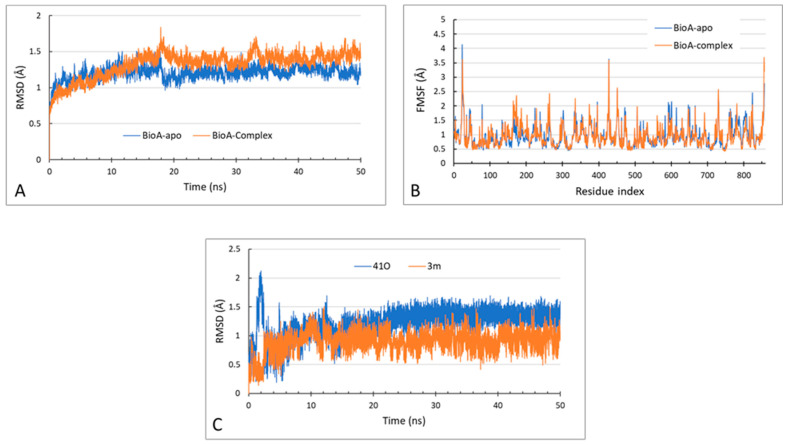
(**A**,**B**) are RMSD and RMSF plots for the simulated BioA-**3m** complex compared to the apo form. (**C**). The RMSD plots for compound **3m** compared to 41O as the simulated system evolved with time.

Figure 3 shows the RMSD and RMSF values for the simulated systems. The average RMSD values of the BioA complex (with both 41O and compound **3m**) was 1.33 Å, which is a little higher than that of the BioA-apo system which showed an RMSD value of 1.20 Å (Figure 3A), which could possibly be attributed to slight conformational changes in the BioA complex to better accommodate the hosted ligands. In addition, the calculated RMSF for each of the two simulated systems, after being fitted to the first frame from the production phase in order to eliminate the effect of rotational and translational motion, showed similar fluctuation patterns with close values (Figure 3B). The average RMSF values for BioA complex and BioA-apo were 0.96 and 0.98 Å, respectively, indicative of stable systems throughout the simulation time.

**Figure 4:**
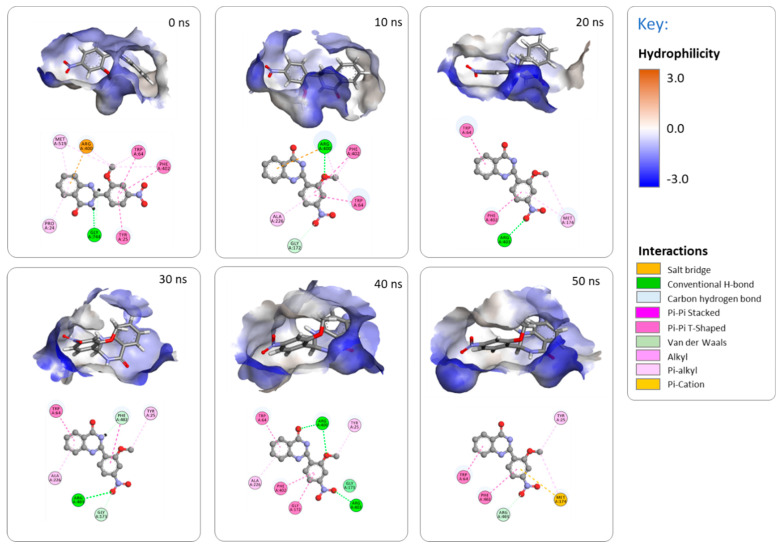
The 3D snapshots of the simulated BioA-**3m** complex at different time points along with their respective 2D interaction maps. Compound **3m** is shown in balls and sticks. The 2D interaction maps show the interactions of compound **3m** with BioA binding site. Coloring is as in Figure 2.

In order to have a closer look at the dynamical behavior of the complexed ligands (41O and **3m**), we calculated their respective RMSD values throughout the simulation time (Figure 3C). The two compounds fluctuated during the first 10 ns until they reached a stable state that was maintained to the end of the 50 ns of simulation. The average RMSD value of compound **3m** was 0.90 Å compared to 1.22 Å for 41O, indicative of stable and good binding affinity. All of the above-discussed dynamical findings can be visually traced in the generated trajectories (Supplementary Materials; Movie S1). Further, snapshots of the simulated BioA complex are shown in Figure 4.

Figure 4 represents different snapshots of the simulated BioA-**3m** complex at different time points. As stated earlier, among the active site residues, Tyr25, Trp64, Trp65, Tyr157, Arg400, and Phe402 were reported to be of high relevance to ligand binding. Compound **3m** has maintained numerous interactions with most of those residues throughout the simulation time, namely Tyr25, Trp64, Arg400, and Phe402. Interactions of **3m** with the BioA active site’s residues included hydrogen bonding between Arg400 and the oxygens of the dihydroquinazolinone and the methoxy, and between Arg403 and the nitro oxygens; Pi-Pi stacking between Trp64 and Phe402 with the phenyl rings; Pi-cation interaction between Arg400 and the phenyl ring of dihydroquinazolinone; in addition to other hydrophobic interactions (see Figure 4 for more details). These stabilizing interactions are seemingly indicative of favorable binding between **3m** and the BioA enzyme. The collective computational results support the conclusion that the BioA enzyme is the most likely target for the tested compounds.

#### 2.3.4. ADME and Toxicity Predictions

In any drug discovery project, it is highly recommended to estimate the ADMET properties for the compounds of interest in order to direct optimization efforts on those that show favorable properties. Therefore, since the anti-TB activities of the tested compounds were promising, their ADMET properties were calculated using the ADMET Descriptors and the Toxicity Prediction (TOPKAT) protocols in DS (Table 3 and Table 4). 

The calculated properties indicated promising ADMET profiles for the tested compounds. All compounds showed good ADME properties, except for the hepatotoxicity descriptor, which showed that all of them were predicted to be hepatotoxic except for compound **3e**. Similarly, all toxicity parameters were favorable, except for mutagenicity predictions of which only compounds **3j** and **3k** showed low potential for mutagenicity. Moreover, their drug-likeness and oral bioavailability were predicted by consideration of Lipinski’s rule of five and Veber’s rule. Lipinski’s rule states that for an orally active drug, no more than one of the following criteria can be violated, namely no more than five hydrogen bond donors, no more than 10 hydrogen bond acceptors, molecular mass of less than 500 daltons, and a log *p* value of less than or equal to 5. For Veber, the rule relies on molecular flexibility, rather than molecular weight, which is accounted for by rotatable bonds that need to be fewer than 10; and on polar surface aria which should be less than 140 Å^2^ (or a total H-bond count fewer than 12) [76]. None of the designed compounds violated any of these rules. According to the above predictions, the designed compounds showed promising ADMET and drug-like properties, and consequently, the active members among the set can be considered candidates for further optimization cycles.

## 3. Materials and Methods

### 3.1. General

All the chemicals and solvents were obtained from Sigma-Aldrich/Merck (St. Louis, MO, USA). To monitor the progress of the chemical reaction, thin-layer chromatography (TLC) was utilized on Merck 60 F-254 silica gel plates using ethyl acetate and n-hexane (6:4) as a solvent system and UV light for visualization. A Buchi melting point B-545 equipment was used to determine melting points. Nicolet 6700 FT-IR spectrometer was used to record the FT-IR spectra. DMSO-*d*_6_ solvent was used to record ^1^H and ^13^C-NMR spectra on Bruker AVANCE III 400 MHz instrument. Chemical shifts (d) were indicated in parts per million downfield from tetramethylsilane, and the coupling constant (*J*) is recorded in Hertz. The splitting pattern is abbreviated as follows: s, singlet; d, doublet; t, triplet; m, multiplet. Mass spectra were recorded using LC-MS-Agilent 1100 series with MSD (Ion trap) using 0.1% aqueous trifluoroacetic acid in acetonitrile system on C18-BDS column. Elemental analysis was performed on Thermo Finnigan FLASH EA 1112 CHN analyzer.

### 3.2. Chemistry

**Scheme 1:**
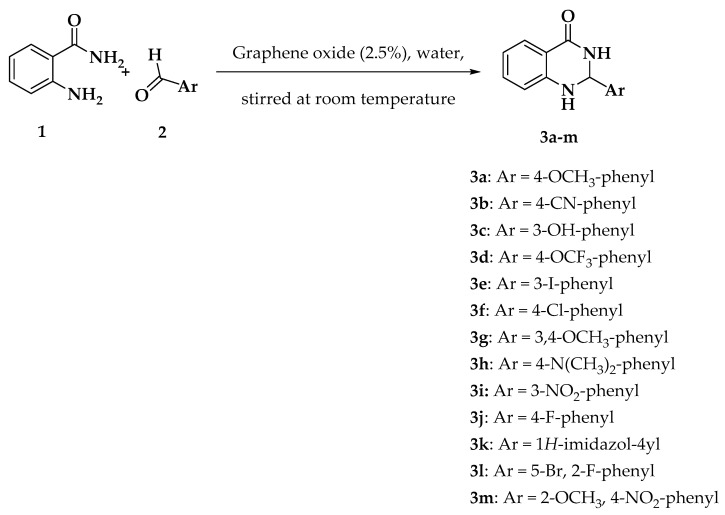
The chemical synthetic scheme for the synthesis of 2,3-dihydroquinazolin-4(1*H*)-one derivatives (**3a**–**m**).

The synthetic scheme for the construction of title compounds is illustrated in Scheme 1. The detailed characterization of the title compounds **3a**–**3k** and **3m**, including single-crystal X-ray data of a representative example (compound **3g**), was described in our previous communication [77,78] with their yield between 82–95%. The scheme of synthesis including instrumental techniques such as FT-IR, ^1^H, and ^13^C-NMR data and spectra of compound **3l** are available as electronic supplementary information (Scheme S1, Figure S1–S3).

### 3.3. Antitubercular Activity

The antitubercular property of the test compounds **3a**–**3m** was evaluated against two types of *M. tuberculosis* strains, namely, H37Rv and well-characterized MDR strains, using the colorimetric Resazurin Microplate Assay (REMA) method as described in our previous communication [79]. The MICs were defined as the minimum drug concentration required to inhibit the organism from growing while leaving no color changes in the well [51]. The MTB reference strain H37Rv (American Type Culture Collection (ATCC) 25177) and MDR-TB were cultured for a total of 3 weeks in Middlebrook 7H11 medium [80] and were then supplemented with Oleic Albumin Dextrose Catalase (OADC) (0.005% *v*/*v* oleic acid, 0.2% *w*/*v* glucose, 0.085% *w*/*v* NaCl, 0.02% *v*/*v* catalase, and 0.5% 171 *w*/*v* bovine serum albumin (BSA)). Incubation was set at 37 °C. The obtained cultures were utilized to prepare an inoculum in a sterile tube with 0.05% Tween 80 and 4.5 mL of phosphate buffer with glass beads (5 mm in diameter) by vortexing. After this, the cultures settled for a total of 45 min; the clear bacterial supernatant was standardized to McFarland Number 1 using sterile water. The resulting bacterial concentration was approximately 1 × 10^7^ colony-forming units (CFU)/mL, which was then diluted with sterile water. Overall, 100 µL of the dilution was added to Middlebrook 7H10 agar plates containing 8–0.125 µg/mL of the agent. The test compounds (8 µg/mL) were dissolved in distilled water and diluted to the required concentration before being added to the agar medium. The test compound MICs were read 3 weeks following 37 °C incubation and were regarded as the minimum drug concentration that could inhibit >99% growth of the bacterial culture when compared to controls.

### 3.4. Cell Line

The cell line under investigation was normal human skin fibroblast cells (CCD-1064SK) purchased from American Type Culture Collection (ATCC) (Manassas, VA, USA). Fibroblast cells were cultured in Iscove’s Modified Dulbecco’s Medium (Euro Clone, Italy) as recommended by ATCC. The media was supplemented with 10% heat-inactivated fetal bovine serum (FBS) (Ebsdorfergrund, Germany), 1% of 2 mM L-glutamine, 100 U/mL penicillin, and 100 µg/mL streptomycin. According to the cells’ growth profile, fibroblast-seeding density was 1 × 10^5^ cell/well. Cell viability was determined by trypan blue exclusion using a hemocytometer.

### 3.5. MTT Cytotoxicity Assay

The MTT colorimetric assay was used to assess the effect of the synthesized compounds on the viability of the fibroblast cell lines. Initially, cells were washed with phosphate buffer saline (PBS) followed by decantation of PBS and cells detachment with 0.25% trypsin-EDTA (Euro Clone). A volume of 10 mL of the culture media was added, and the cell suspension was centrifuged at 1000 rpm for 10 min. The pellets were resuspended in a 10 mL medium to make a single-cell suspension. The viability of the cells was determined by trypan blue exclusion, and it exceeded 90% as counted in a hemocytometer. The cell suspension was diluted to give the optimal seeding density, and 100 µL of the cell suspension was plated in a 96-well plate. Cells were cultured at 37 °C in a humidified atmosphere of 5% CO_2_. After 24 h incubation, the cells were treated with 100 µM of the synthesized compounds (diluted in culture media to yield the required concentration) and then incubated for 72 h. At the end of the exposure time, 15 µL of MTT stock solution (5 mg/mL in sterile PBS, pH 7.4) (Promega, Madison, WI, USA) was added to each well and incubated for 3 h. After that, 100 µL of solubilizing stop solution was added to each well to solubilize the dark violet formazan crystals. The optical densities at 570 nm were then measured using a micro-plate reader (Biotek, Winooski, VT, USA), and the percentage of cell viability was calculated with respect to a control corresponding to untreated cells.

### 3.6. Computational Methods

**Figure 5:**
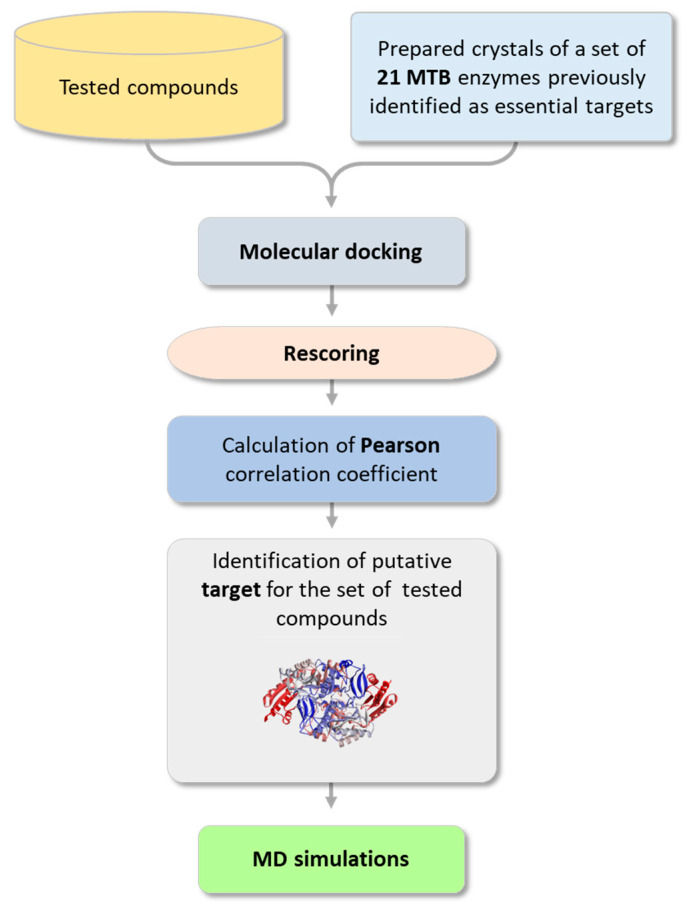
Summary of the computational workflow implemented in this study.

The same computational approach we implemented previously to identify the putative mycobacterial targets for the screened compounds was used in this study [52] (Figure 5). Briefly, an extensive literature review revealed 48 macromolecular targets that are essential for mycobacterial survival, of which 21 have solved 3D crystal structures (deposited in the Protein Data Bank) that were used in molecular docking studies [81,82,83]. All crystal structures were prepared, solvated, and minimized using Biovia Discovery Studio 2020 [52,84]. Then, the tested compounds were also prepared using DS and were docked into the binding sites of the 21 proteins using the CDOCKER algorithm in DS. All docked poses were then rescored using different scoring functions, and their Pearson correlation coefficients with the experimental MIC values were calculated. The enzyme with the highest correlation value was identified as a putative target for the tested compounds. Finally, the binding mode and binding stability of a virtual complex of compound **3m** with its putative target were simulated using Amber 12 relative to the native co-crystallized inhibitor. Moreover, a range of pharmacokinetic and toxicity descriptors for the tested compounds (**3a**–**3m**) was calculated using the ADMET Descriptors protocol and the Toxicity Prediction (TOPKAT) protocol in DS [85].

## 4. Conclusions

This research is part of our ongoing attempts to find potential new anti-TB agents, where a series of substituted 2,3-dihydroquinazolin-4(1*H*)-one analogues (**3a**–**3m**) was evaluated for their anti-MTB activity (in vitro) against the drug-susceptible H37Rv and MDR strains of MTB. The MIC values of the compounds showed good anti-MTB inhibitory activities ranging from 2-128 µg/mL. Compounds **3l** and **3m** attached with di-substituted aryl moiety (having electron withdrawing halogens) at the 2-position of the quinazoline scaffold were the most active, with a MIC value of 2 µg/mL against the drug-susceptible H37Rv strain of MTB. Compound **3k** also showed significant inhibitory activity against both the H37Rv and MDR strains with MIC values of 4 and 16 µg/mL, respectively. The presence of the imidazole ring at the 2-position of the quinazoline scaffold probably resulted in distinguished inhibitory activity for compound **3k** against the MDR strain, whereas other analogues did not show any inhibitory activity against the MDR strain of MTB. The safety and tolerability of the compounds **3a**–**3m** were evaluated by carrying out the in vitro MTT assay against the normal human skin fibroblast cells, where the most active compounds **3l** and **3m** were found to be tolerable with 36% and 30% toxicity, respectively.

Computational studies were also carried out to identify the putative target for the tested compounds (**3a**–**3m**). The computational results revealed the mycobacterial BioA enzyme as the most likely putative target. Collectively, the good biological results, the promising ADMET descriptors, and the identification of a putative target are encouraging to conduct further optimization of the most active lead molecules **3k**–**3m.** Moreover, the current findings highlighted the importance of experimentally validating the identified putative target to develop 2,3-dihydroquinazolin-4(1*H*)-ones as probable BioA inhibitors in order to combat the drug-sensitive and drug-resistant strains of MTB.

## Figures and Tables

**Table 1:** In vitro whole-cell anti-TB results of substituted 2,3-dihydroquinazolin-4(1*H*)-ones analogues (**3a**–**m**) against *Mycobacterium tuberculosis* and their cytotoxic effect on fibroblast cells.

Compound Code	Compound Structure	Level of Activity (µg/mL)		Cytotoxicity (%) at 100 µM *
		Susceptible (H37Rv)	MDR	
**3a**	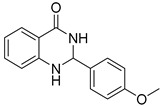	64	>128	28.8 ± 0.6
**3b**	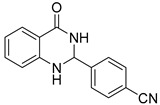	>64	No activity	19.5 ± 5.0
**3c**	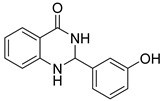	8	>32	58.5 ± 1.1
**3d**	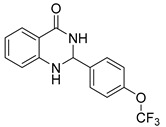	>64	No activity	47.6 ± 1.6
**3e**	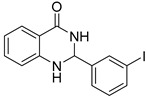	128	No activity	72.0 ± 8.3
**3f**	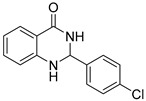	>128	No activity	57.6 ± 3.7
**3g**	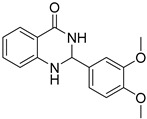	64	No activity	32.5 ± 11.0
**3h**	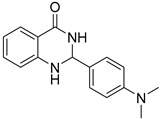	>128	No activity	54.6 ± 2.8
**3i**	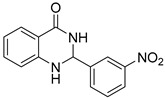	>64	No activity	24.1 ± 3.9
**3j**	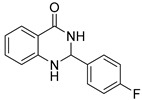	>64	No activity	44.1 ± 11.5
**3k**	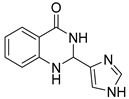	4	16	19.2 ± 4.9
**3l**	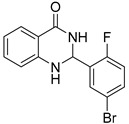	2	>64	36.3 ± 4.2
**3m**	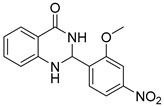	2	>64	30.9 ± 8.8

* Values represent the mean ± SD of three replicates.

**Table 2:** The overall correlation coefficients matrix between the minimum inhibitory concentration values of the tested compounds and their computational scores obtained from different scoring functions for each of the selected target enzymes.

Index	Name	LS1	LS2	PLP1	PLP2	Jain	PMF	PMF4	CDE	CDIE	Ludi-1	Ludi-2	Ludi-3
1	Alanine racemase	−0.71	0.29	−0.16	−0.12	0	−0.06	−0.51	0.38	0.31	−0.15	−0.19	−0.35
2	CamA	−0.57	−0.39	−0.12	−0.05	−0.09	−0.07	−0.45	−0.3	−0.38	0.44	0.19	−0.06
3	DprE1	−0.3	0.62	0.1	−0.01	−0.12	0.28	−0.82	0.51	0.38	0.33	0.2	0.3
4	FabH	−0.67	0.26	−0.42	−0.44	−0.02	−0.24	−0.75	0.35	0.32	−0.24	−0.3	−0.31
5	InhA	−0.59	−0.5	0.12	0.32	0.32	0.12	−0.29	0.02	−0.08	0.4	0.49	−0.05
6	MabA	−0.4	0.35	−0.3	−0.23	−0.51	−0.46	−0.66	0.28	0.16	−0.13	−0.15	−0.34
7	AspS	−0.68	−0.32	−0.14	−0.05	0.05	0.08	−0.51	0.06	−0.16	0.06	0.01	−0.03
8	LeuRS	−0.82	0.54	0.3	−0.06	−0.15	−0.27	−0.62	0.41	0.08	0.58	0.54	0.13
9	GlmU	−0.3	0.52	−0.32	0.11	−0.36	0.05	−0.81	0.43	0.2	0.17	0.17	0.33
10	PanK	−0.02	0.75	−0.14	−0.12	−0.08	−0.15	−0.31	0.25	0.25	0.56	0.38	0.08
11	PknB	−0.36	−0.58	−0.56	−0.3	0.03	−0.03	−0.67	0.03	−0.2	0.1	0.12	−0.12
12	PknA	−0.42	0.4	−0.07	0.11	−0.76	0.13	−0.52	0.17	0.01	0.25	0.05	0.03
13	KasA	−0.28	−0.08	−0.62	−0.48	−0.38	−0.24	−0.38	−0.04	−0.26	−0.27	−0.25	−0.43
14	KasA (BS2) *	−0.27	0.38	0.17	0.06	0.32	0.43	−0.44	0.14	0.43	−0.02	0.06	0.16
15	Pks13	−0.1	0.32	0.3	0.39	0.66	−0.12	−0.66	0.59	0.29	0.1	0.22	0.25
16	BioA ^#^	−0.63	0.1	0.14	0.28	0.65	−0.42	−0.86	0.74	0.55	0.52	0.58	0.65
17	EchA6	−0.33	0.17	−0.04	0.3	0.31	−0.17	−0.69	0.38	0.31	0.46	0.05	0.18
18	MmpL3	−0.77	−0.38	0.25	0.31	0.14	0.32	−0.39	0.04	−0.07	0.1	0.1	0.42
19	AspAT	−0.41	0.67	−0.24	0.24	0.16	0.01	−0.62	0.23	0.14	0.15	0.41	0.44
20	MurE	−0.83	−0.05	0.25	0.35	0.6	−0.31	−0.38	0.31	0.15	0.33	0.29	0.2
21	EthR	−0.39	0.17	0.06	0.19	−0.41	−0.24	−0.78	0.48	0.13	0.13	0.22	0.12
22	PrpC	−0.4	0.29	−0.44	−0.46	0.08	0.04	−0.56	0.37	0.3	0.08	0.16	0.05

* KasA enzyme has two binding sites; an acyl chain-binding site (BS1), and the catalytic active site (BS2). Both were used for docking. This refers to the second binding site. ^#^ The highlighted values, in bold red, represent the highest obtained correlation coefficient.

**Table 3:** Calculated ADME descriptors.

Compounds		MIC (µg/mL)		ADME Descriptors *								
Index	Code	H37Rv	MDR-MTB	AS	BBB	CYP2D6 Inhibition		Hepatotoxicity	HIA ^a^	PPB ^b^	AlogP	PSA ^c^
1	3a	64	>128	3	2	FALSE		TRUE	0	TRUE	2.432	51.851
2	3b	>64	No activity	3	2	FALSE		TRUE	0	TRUE	2.327	65.856
3	3c	8	>32	3	2	FALSE		TRUE	0	TRUE	2.206	63.736
4	3d	>64	No activity	1	1	FALSE		TRUE	0	TRUE	4.568	51.851
5	3e	128	No activity	2	1	FALSE		FALSE	0	TRUE	3.026	42.921
6	3f	>128	No activity	2	1	FALSE		TRUE	0	TRUE	3.113	42.921
7	3g	64	No activity	3	2	FALSE		TRUE	0	TRUE	2.415	60.781
8	3h	>128	No activity	3	2	FALSE		TRUE	0	TRUE	2.61	46.273
9	3i	>64	No activity	3	3	FALSE		TRUE	0	TRUE	2.343	85.744
10	3j	>64	No activity	3	2	FALSE		TRUE	0	TRUE	2.654	42.921
11	3k	4	16	4	3	FALSE		TRUE	0	FALSE	0.666	73.031
12	3l	2	>64	2	1	FALSE		TRUE	0	TRUE	3.402	42.921
13	3m	2	>64	2	3	FALSE		TRUE	0	TRUE	2.326	94.674
**Aqueous Solubility (AS)**						**BBB Penetration**		**Human Intestinal Absorption (HIA)**				
Level		Drug-likeness				Level	Description	Level	Description			
0		Extremely low				0	Very High	0	Good absorption			
1		No, very low, but possible				1	High	1	Moderate absorption			
2		Yes, low				2	Medium	2	Low absorption			
3		Yes, good				3	Low	3	Very low absorption			
4		Yes, optimal				4	Undefined					
5		No, too soluble										

^a^: human intestinal absorption, ^b^: plasma protein binding, ^c^: polar surface area. * Key to the above-calculated ADME descriptors.

**Table 4:** Calculated toxicity parameters.

Compounds		MIC (µg/mL)		Toxicity Parameters *								
Index	Code	H37Rv	MDR-MTB	AM ^a^	SI ^b^	OI ^c^	AB ^d^	DTP ^e^	CMR ^f^	CFR	CLM	CFM
1	3a	64	>128	0.997	0.016	0	0	0.026	0	0	0.988	1
2	3b	>64	No activity	1	0	0	0.001	0	0	0	0	1
3	3c	8	>32	1	0	0	0	0.002	0	0	0.007	1
4	3d	>64	No activity	1	0.219	0	0	0.997	0	0	1	1
5	3e	128	No activity	1	0	0	0.003	0.001	0	0	0.27	1
6	3f	>128	No activity	0.998	0	0	0	0.004	0	0	0.978	1
7	3g	64	No activity	1	0.061	0	0	0.275	0	0	0.956	1
8	3h	>128	No activity	1	0	0	0.969	0	0	0	0	1
9	3i	>64	No activity	1	0	0	0	0.002	0	0	1	1
10	3j	>64	No activity	0.078	0	0	0	0.101	0	0	1	1
11	3k	4	16	0	0	0	0	1	0	0	1	1
12	3l	2	>64	0.956	0	0	0	0.154	0	0	1	1
13	3m	2	>64	1	0	0	0	0.056	0	0	1	1
**Probability Values**		**Probability Level**		**Description**								
0.0 to 0.30		Low probability		Such a chemical is not likely to produce a positive response in an experimental assay								
>0.30 but <0.70		Intermediate probability										
>0.70		High probability		Likely to produce a positive response in an experimental assay								

^a^: Ames mutagenicity, ^b^: skin irritation, ^c^: ocular irritancy, ^d^: Aerobic Biodegradability, ^e^: Developmental toxicity potential, ^f^: rodent carcinogenicity (for male and female rats and mice, CMR, CFR, and CLM, CFM, respectively). * Key to the above-calculated toxicity parameters.

## Data Availability

Not applicable.

## Supplementary Materials

The following supporting information can be downloaded at: https://www.mdpi.com/article/10.3390/antibiotics11070831/s1, Scheme S1: Synthetic scheme, procedure, characterization details of 2-(5-bromo-2-fluorophenyl)-2,3-dihydroquinazolin-4(1*H*)-one (**3l**); Figure S1: FT-IR of 2-(5-bromo-2-fluorophenyl)-2,3-dihydroquinazolin-4(*1H*)-one (**3l**); Figure S2: ^1^H-NMR of 2-(5-bromo-2-fluorophenyl)-2,3-dihydroquinazolin-4(1*H*)-one (**3l**); Figure S3: ^13^C-NMR of 2-(5-bromo-2-fluorophenyl)-2,3-dihydroquinazolin-4(1*H*)-one (**3l**); Table S1: Reported essential and potential mycobacterial drug targets. Table S2: The selected 20 essential mycobacterial drug targets that we used for molecular modeling studies. References [86,87,88,89,90,91,92,93,94,95,96,97,98,99,100,101,102,103,104,105,106,107,108,109,110,111,112,113,114,115,116,117,118,119] are cited in the Supplementary Materials.
