# Supplementary material for: Antitubercular, Cytotoxicity, and Computational Target Validation of Dihydroquinazolinone Derivatives
Source: Antibiotics (Basel). 2022 Jun 21;11(7):831. doi: 10.3390/antibiotics11070831 (PMC9311641; doi:10.3390/antibiotics11070831)
Supplement: Supplementary file 1 [file antibiotics-11-00831-s001.zip › antibiotics-1770650-supplementary.pdf]

Supplementary materials

Antitubercular, Cytotoxicity, and Computational Target Validation of Dihydroquinazolinone Derivatives

Katharigatta N. Venugopala [1,2,*], Nizar A. Al-Shar'I [3], Lina A. Dahabiyeh [4], Wafa Hourani [5], Pran Kishore Deb [5,*], Melendhran Pillay [6], Bashaer Abu-Irmaileh [7], Yasser Bustanji [7,8], Sandeep Chandrashekharappa [9], Christophe Tratrat [1], Mahesh Attimarad [1], Anroop B. Nair [1], Nagaraja Sreeharsha [1,10], Pottathil Shinu [11], Michelyne Haroun [1], Mahmoud Kandeel [12,13], Abdulmalek Ahmed Balgoname [1], Rashmi Venugopala [14] and Mohamed A. Morsy [1,15]

[1] Department of Pharmaceutical Sciences, College of Clinical Pharmacy, King Faisal University, Al-Ahsa 31982, Saudi Arabia; ctratrat@kfu.edu.sa (C.T.); mattimarad@kfu.edu.sa (M.A.); anair@kfu.edu.sa (A.B.N.); sharsha@kfu.edu.sa (N.S.); mharoun@kfu.edu.sa (M.H.); abalgoname@kfu.edu.sa (A.A.B.); momorsy@kfu.edu.sa (M.A.M.)

[2] Department of Biotechnology and Food Science, Faculty of Applied Sciences, Durban University of Technology, Durban 4000, South Africa

[3] Department of Medicinal Chemistry and Pharmacognosy, Faculty of Pharmacy, Jordan University of Science and Technology, P.O. Box 3030, Irbid, 22110, Jordan; nashari@just.edu.jo (N.A.A.-I.)

[4] Department of Pharmaceutical Sciences, School of Pharmacy, The University of Jordan, 11942 Amman, Jordan; l.dahabiyeh@ju.edu.jo (L.A.D.)

[5] Department of Pharmaceutical Sciences, Faculty of Pharmacy, Philadelphia University, Amman 19392, Jordan; wafa.hourani@hotmail.com (W.H.)

[6] Department of Microbiology, National Health Laboratory Services, KZN Academic Complex, Inkosi Albert Luthuli Central Hospital, Durban 4001, South Africa; melendhra.pillay@nhls.ac.za (M.P.)

[7] Hamdi Mango Center for Scientific Research, The University of Jordan, 11942 Amman, Jordan; abuirmailehbashaer@yahoo.com (B.A.-I); bustanjiyasser@gmail.com (Y.B.)

[8] Department of Basic Medical Sciences, College of Medicine, University of Sharjah, Sharjah 27272, UAE

[9] Department of Medicinal Chemistry, National Institute of Pharmaceutical Education and Research (NIPER-R) Raebareli, Lucknow UP 226002, India; c.sandeep@niperraebareli.edu.in (S.C.)

[10] Department of Pharmaceutics, Vidya Siri College of Pharmacy, Off Sarjapura Road, Bangalore 560035, India

[11] Department of Biomedical Sciences, College of Clinical Pharmacy, King Faisal University, Al-Ahsa 31982, Saudi Arabia; spottathail@kfu.edu.sa (P.S.)

[12] Department of Biomedical Sciences, College of Veterinary Medicine, King Faisal University, Al-Ahsa 31982, Saudi Arabia; mkandeel@kfu.edu.sa (M.K.)

[13] Department of Pharmacology, Faculty of Veterinary Medicine, Kafrelsheikh University, Kafrelsheikh 33516, Egypt

[14] Department of Public Health Medicine, Howard College Campus, University of KwaZulu-Natal, Durban 4001, South Africa; rashmivenugopala@gmail.com (R.V.)

[15] Department of Pharmacology, Faculty of Medicine, Minia University, El-Minia 61511, Egypt

* Correspondence: kvenugopala@kfu.edu.sa (K.N.V.); prankishore1@gmail.com (P.K.D.)

Table of contents

Sl. No.	Particulars	Page Number
01	**Scheme S1.** Synthetic scheme, procedure, characterization details of 2-(5-bromo-2-fluorophenyl)-2,3-dihydroquinazolin-4(1*H*)-one (**3l**)	3
02	**Figure S1:** FT-IR of 2-(5-bromo-2-fluorophenyl)-2,3-dihydroquinazolin-4(1*H*)-one (**3l**)	4
03	**Figure S2:** ^{1}H-NMR of 2-(5-bromo-2-fluorophenyl)-2,3-dihydroquinazolin-4(1*H*)-one (**3l**)	5
04	**Figure S3:** ^{13}C-NMR of 2-(5-bromo-2-fluorophenyl)-2,3-dihydroquinazolin-4(1*H*)-one (**3l**)	6
05	**Table S1:** Reported essential and potential mycobacterial drug targets.	7
06	**Table S2:** The selected 20 essential mycobacterial drug targets that we used for molecular modeling studies.	9
07	References	9

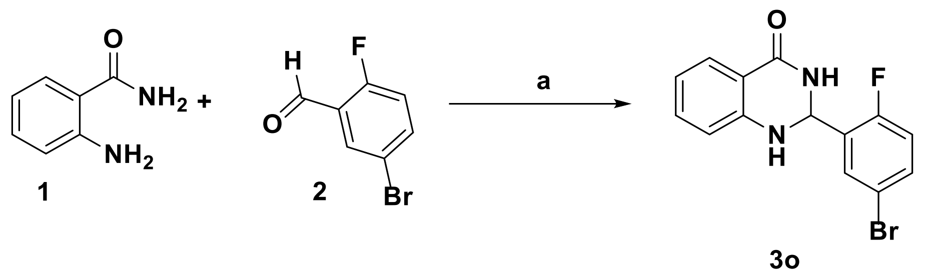

Scheme S1. Synthetic scheme for the preparation of 2-(5-bromo-2-fluorophenyl)-2,3-dihydroquinazolin-4(1*H*)-one (**3l**). Reagents and conditions: (**a**) graphene oxide (2.5%), water, stirred at room temperature; yield 82%–95%.

Synthetic procedure for 2-(5-bromo-2-fluorophenyl)-2,3-dihydroquinazolin-4(1*H*)-one (3l)

A mixture of 5-bromo-2-fluorobenzaldehyde (1.5 mmoL) and 2-aminobenzamide (1 mmoL) in 10 mL of water was stirred in a round-bottom flask with graphene oxide (2.5%) at room temperature, as depicted in Scheme S1. The progress of the reaction was monitored with TLC. After completion of the reaction, the insoluble precipitate obtained was separated and filtrated, and the crude product was washed with 50% cold ethanol. The crude product was recrystallized from 75% ethyl alcohol to obtain 65% of the title compound 2-(4-fluorophenyl)-2,3-dihydroquinazolin-4(1*H*)-one at 98% purity.

FT-IR (KBr, cm^{-1}) 3303, 3232, 3195, 3076, 2920, 1650, 1612, 1512, 1477, 1396, 1251, 1157, 821, 750. ^{1}H-NMR (400 MHz,, DMSO-d_6) δ = 8.45 (1H, s), 7.83-7.81 (1H, m), 7.66-7.59 (2H, m), 7.30-7.13 (3H, m), 6.84-6.71 (2H, m), 6.05 (1H, s); ^{13}C-NMR (100 MHz, DMSO-d_6) δ= 164.56, 158.36, 157.78, 147.37, 134.55, 133.55, 133.23, 131.05, 130.58, 126.44, 118.72, 118.13, 116.00, 115.48, 114.46. Anal. calculated for $C_{14}H_{10}BrFN_2O$: C, 52.36; H, 3.14; N, 8.72; found C, 52.27, H, 3.19; N, 8.68.

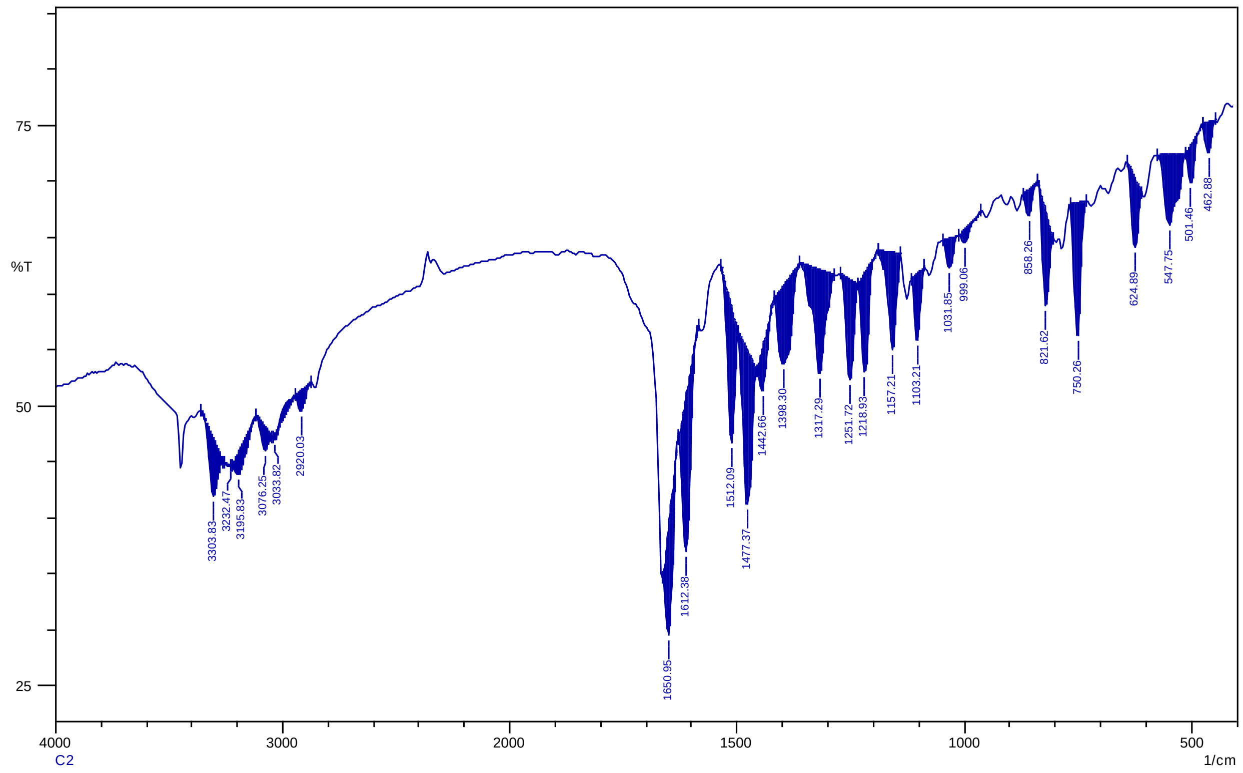

Figure S1: FTIR of 2-(5-Bromo-2-fluorophenyl)-2,3-dihydroquinazolin-4(1*H*)-one (**3l**)

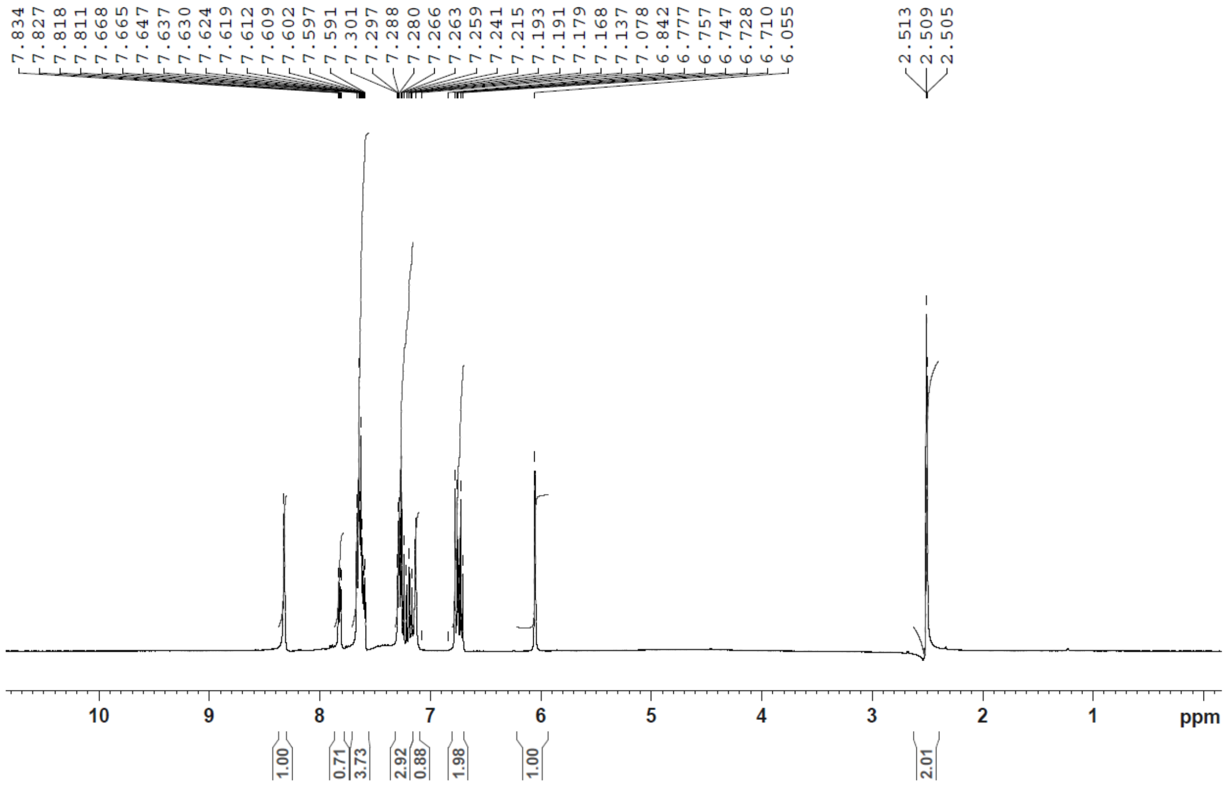

Figure S2: ^{1}H-NMR of 2-(5-bromo-2-fluorophenyl)-2,3-dihydroquinazolin-4(1*H*)-one (**3l**)

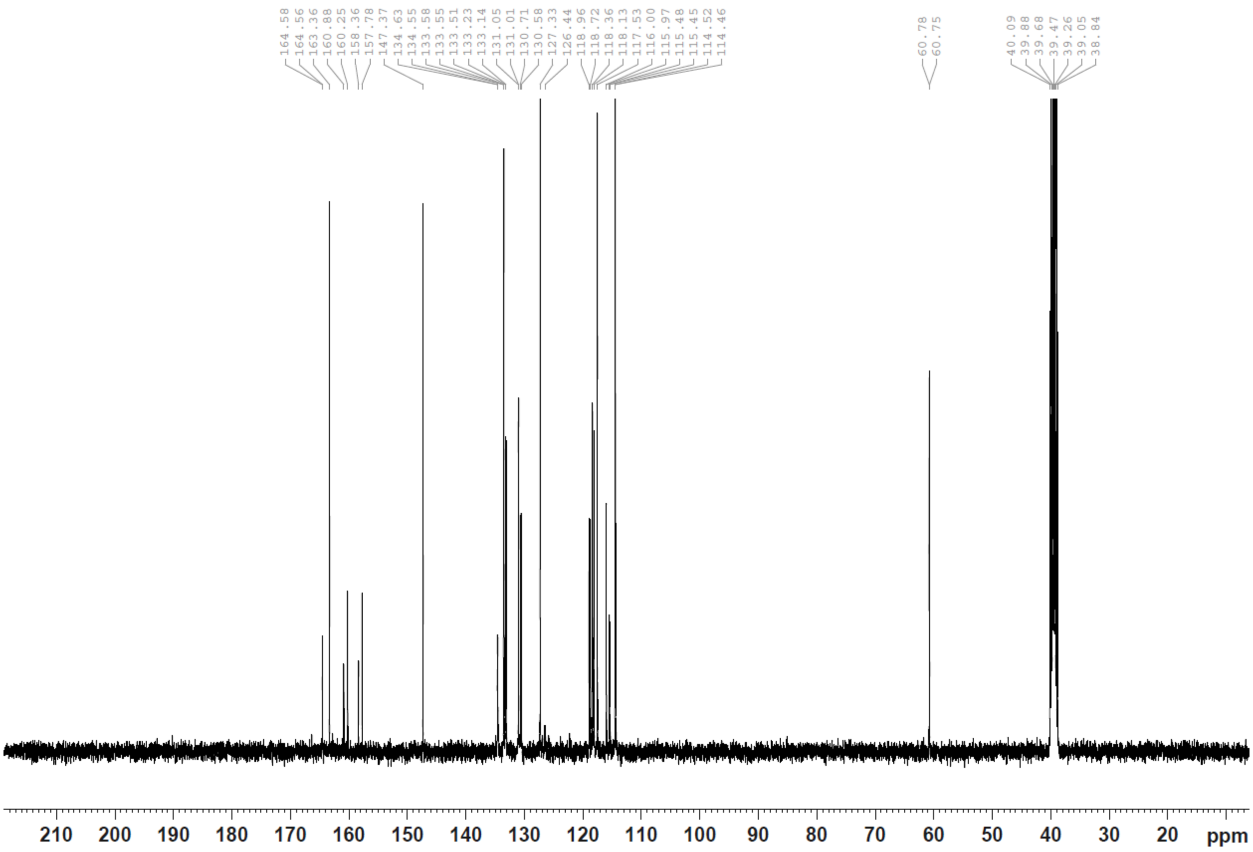

Figure S3: ^{13}C-NMR of 2-(5-bromo-2-fluorophenyl)-2,3-dihydroquinazolin-4(1*H*)-one (**3l**)

Table S1: Reported essential and potential mycobacterial drug targets.

Index	MTB Protein Target	Targeted pathway	Availability of 3D structure	References
1	Decaprenylphosphoryl-β-d-ribofuranose oxidoreductase (DprE1)	Cell wall synthesis: arabinogalactan biosynthesis	Yes	[1-3]
2	Enyol-ACP-reductase, (InhA)	Cell wall synthesis: mycolic acid biosynthesis	Yes	[4]
3	Mycolic acid cyclopropane synthase (CmaA2)	Cell wall synthesis: mycolic acid biosynthesis	Yes	[5]
4	Alanine racemase (alr)	Cell wall biosynthesis: peptidoglycan biosynthesis	Yes	[6]
5	3-oxoacyl-[acyl-carrier-protein] synthase 3 (FabH)	Fatty acid biosynthesis	Yes	[7]
6	β-ketoacyl-ACP reductase (MabA)	Fatty Acid Biosynthesis	Yes	[8]
7	Aspartyl-tRNA Synthetase (AspS)	Protein synthesis	Yes	[9]
8	leucyl-tRNA synthase (LeuRS)	Protein synthesis	Yes	[10]
9	Bifunctional enzyme (GlmU)	Cell wall biosynthesis	Yes	[11]
10	Pantothenate kinase (PanK, type 1)	Cofactor biosynthesis: Coenzyme A biosynthesis	Yes	[12]
11	Protein kinase B (PknB)	Signal transduction	Yes	[13]
12	Protein kinase A (PknA)	Signal transduction	Yes	[14]
13	β-ketoacyl acyl carrier protein synthase I (KasA)	Cell wall synthesis: mycolic acid biosynthesis	Yes	[7]
14	Polyketide synthase (Pks13)	Cell wall synthesis: mycolic acid biosynthesis	Yes	[15, 16]
15	5′-pyridoxal phosphate (PLP)-dependent aminotransferase (BioA)	Cofactor biosynthesis: biotin biosynthesis	Yes	[17]
16	Aspartate aminotransferase (aspAT)	Asp biosynthesis, and Asp-dependent nitrogen metabolism	Yes	[18]
17	Enoyl-CoA hydratase 6 (EchA6)	Cell wall synthesis: mycolic acid biosynthesis	Yes	[19]
18	Transcriptional repressor of EthA monooxygenase (EthR)	Cell wall synthesis: mycolic acid biosynthesis (indirect)	Yes	[20]
19	MurE (Mur Ligase family)	Cell wall biosynthesis: peptidoglycan biosynthesis	Yes	[21]
20	2-methylcitrate synthase (PrpC) or (GltA3)	Fatty Acid Biosynthesis	Yes	[22]
21	Transmembrane transport protein large (MmpL3)	Cell wall synthesis: mycolic acid biosynthesis	Yes	[2]
22	CTP synthetase (PyrG)	DNA synthesis	Yes	[23]
23	3-oxoacyl-(Acyl-carrier-protein) reductase FabG4 (FabG-1)	Fatty Acid Biosynthesis	Yes	[24]
24	Triosephosphate isomerase (TpiA)	Gluconeogenetic pathways	Yes	[25]
25	Serine hydroxymethyltransferase 1 (GlyA1)	Tetrahydrofolate interconversion	Yes	[26]
26	Glycine cleavage system H protein (gcvH)	Degradation of glycine	Yes	[24]
27	6-phosphogluconolactonase (pgl) or (devB)	Carbohydrate degradation	Yes	[24]
28	O-succinylhomoserine sulfhydrylase (metZ)	Amino-acid biosynthesis	Yes	[24]

29	S-adenosylmethionine synthetase (metK)	Amino-acid biosynthesis	Yes	[24]
30	Thymidylate synthase (ThyX)	Pyrimidine metabolism	Yes	[27]
31	steroid-degrading 9lavin monooxygenase (HsaA) and (HsaB)	Cholesterol metabolism	Yes	[22]
32	phosphatidyl-myoinositol mannosyltransferase (PimA)	Cell wall synthesis	No, M. seg.	[28]
33	DNA topoisomerases and DNA gyrase	DNA replication	Yes	[29]
34	Malate synthase (GlcB)	Fatty acid metabolism: The glyoxylate shunt	Yes	[30]
35	D-alanine:D-alanine Ligase	Cell wall biosynthesis: peptidoglycan biosynthesis	Yes	[31]
36	Arabinosyltransferase C, A, and B (embC); (embA); (embB)	Cell wall synthesis: arabinogalactan biosynthesis	Yes	[32, 33]
37	Phospho-N-acetylmuramoyl-pentapeptide-transferase (mraY)	Cell wall biosynthesis: peptidoglycan biosynthesis	No	[32]
38	ESX-3 secretion system ATPase (EccB3)	ESX-3 specialized secretion system	No, M. seg.	[15]
39	1,4-dihydroxy-2-naphthoate octaprenyltransferase (MenA)	Energy metabolism	No	[7]
40	Demethylmenaquinone methyltransferase (MenG)	Energy metabolism	No	[7]
41	MoeW	Cofactor biosynthesis: molybdenum biosynthetic	No	[1]
43	Cytochrome b subunit of the cytochrome bc1 complex (QcrB)	Energy metabolism and ATP synthesis	No	[2]
44	Acid resistance periplasmic serine protease (MarP)	Intracellular pH homeostasis	No	[10]
45	Decaprenyl-phosphate N-acetylglucosaminephosphotransferase (wecA)	Cell wall synthesis: arabinogalactan biosynthesis	No	[34]
46	50S ribosomal protein L3 (rplC)	Protein synthesis	No	[32]
47	ATP synthase (atpE)	Energy metabolism	Yes, rotor ring	[2]
48	Dihydrofolate reductase (dfrA)	DNA precursor synthesis	Yes	[10, 35]

Table S2: The selected 20 essential mycobacterial drug targets that we used for molecular modeling studies.

Index	MTB Protein Target	Targeted pathway	PDB ID	References
1	Decaprenylphosphoryl-β-d-ribofuranose oxidoreductase (DprE1)	Cell wall biosynthesis: arabinogalactan biosynthesis	4P8C	[1-3]
2	Enyol-ACP-reductase, (InhA)	Cell wall biosynthesis: mycolic acid biosynthesis	6R9W	[4]
3	Mycolic acid cyclopropane synthase (CmaA2)	Cell wall biosynthesis: mycolic acid biosynthesis	1KPI	[5]
4	β-ketoacyl acyl carrier protein synthase I (KasA)	Cell wall biosynthesis: mycolic acid biosynthesis	6P9L	[7]
5	Polyketide synthase (Pks13)	Cell wall biosynthesis: mycolic acid biosynthesis	5V3Y	[15, 16]
6	Enoyl-CoA hydratase 6 (EchA6)	Cell wall biosynthesis: mycolic acid biosynthesis	5DUF	[19]
7	Transcriptional repressor of EthA monooxygenase (EthR)	Cell wall biosynthesis: mycolic acid biosynthesis (indirect)	5EYR	[20]
8	Alanine racemase (alr)	Cell wall biosynthesis: peptidoglycan biosynthesis	1XFC	[6]
9	MurE (Mur Ligase family)	Cell wall biosynthesis: peptidoglycan biosynthesis	2WTZ	[21]
10	Bifunctional enzyme (GlmU)	Cell wall biosynthesis	2QKX	[11]
11	2-methylcitrate synthase (PrpC) or (GltA3)	Fatty Acid Biosynthesis	3HWK	[22]
12	3-oxoacyl-[acyl-carrier-protein] synthase 3 (FabH)	Fatty acid biosynthesis	1HZP	[7]
13	β-ketoacyl-ACP reductase (MabA)	Fatty Acid Biosynthesis	1UZN	[8]
14	Aspartyl-tRNA Synthetase (AspS)	Protein synthesis	5W25	[9]
15	leucyl-tRNA synthase (LeuRS)	Protein synthesis	5AGS	[10]
16	Protein kinase B (PknB)	Signal transduction	5U94	[13]
17	Protein kinase A (PknA)	Signal transduction	6B2Q	[14]

18	Pantothenate kinase (PanK, type 1)	Cofactor biosynthesis: Coenzyme A biosynthesis	4BFZ	[12]
19	5′-pyridoxal phosphate (PLP)-dependent aminotransferase (BioA)	Cofactor biosynthesis: biotin biosynthesis	4XJO	[17]
20	Aspartate aminotransferase (aspAT)	Asp biosynthesis, and Asp-dependent nitrogen metabolism	6U7A	[18]
21	Transmembrane transport protein large (MmpL3)	Cell wall synthesis: mycolic acid biosynthesis	6AJJ	[2]

References

1. Wang, F., et al., *Identification of a small molecule with activity against drug-resistant and persistent tuberculosis.* Proceedings of the National Academy of Sciences, 2013. **110**(27): p. E2510-E2517.DOI: http://doi.org/10.1073/pnas.1309171110.
2. Torfs, E., et al., *Opportunities for Overcoming Mycobacterium tuberculosis Drug Resistance: Emerging Mycobacterial Targets and Host-Directed Therapy.* International journal of molecular sciences, 2019. **20**(12): p. 2868.DOI: http://doi.org/10.3390/ijms20122868.
3. Piton, J., C.S.Y. Foo, and S.T. Cole, *Structural studies of Mycobacterium tuberculosis DprE1 interacting with its inhibitors.* Drug Discovery Today, 2017. **22**(3): p. 526-533.DOI: http://doi.org/10.1016/j.drudis.2016.09.014.
4. Kamsri, P., et al., *Discovery of New and Potent InhA Inhibitors as Antituberculosis Agents: Structure-Based Virtual Screening Validated by Biological Assays and X-ray Crystallography.* Journal of Chemical Information and Modeling, 2020. **60**(1): p. 226-234.DOI: http://doi.org/10.1021/acs.jcim.9b00918.
5. Huang, C.-c., et al., *Crystal Structures of Mycolic Acid Cyclopropane Synthases fromMycobacterium tuberculosis.* Journal of Biological Chemistry, 2002. **277**(13): p. 11559-11569.DOI: http://doi.org/10.1074/jbc.M111698200.
6. LeMagueres, P., et al., *The 1.9 Å Crystal Structure of Alanine Racemase from Mycobacterium tuberculosis Contains a Conserved Entryway into the Active Site.* Biochemistry, 2005. **44**(5): p. 1471-1481.DOI: http://doi.org/10.1021/bi0486583.
7. Shetye, G.S., S.G. Franzblau, and S. Cho, *New tuberculosis drug targets, their inhibitors, and potential therapeutic impact.* Translational Research, 2020. **220**: p. 68-97.DOI: http://doi.org/10.1016/j.trsl.2020.03.007.
8. Cohen-Gonsaud, M., et al., *Crystal Structure of MabA from Mycobacterium tuberculosis, a Reductase involved in Long-chain Fatty Acid Biosynthesis.* Journal of Molecular Biology, 2002. **320**(2): p. 249-261.DOI: http://doi.org/10.1016/S0022-2836(02)00463-1.
9. Gurcha, S.S., et al., *Biochemical and structural characterization of mycobacterial aspartyl-tRNA synthetase AspS, a promising TB drug target.* PloS one, 2014. **9**(11): p. e113568-e113568.DOI: http://doi.org/10.1371/journal.pone.0113568.
10. Yuan, T. and N.S. Sampson, *Hit Generation in TB Drug Discovery: From Genome to Granuloma.* Chemical Reviews, 2018. **118**(4): p. 1887-1916.DOI: http://doi.org/10.1021/acs.chemrev.7b00602.
11. Chen, C., et al., *The Inhibitory Effect of GlmU Acetyltransferase Inhibitor TPSA on Mycobacterium tuberculosis May Be Affected Due to Its Methylation by Methyltransferase Rv0560c.* Frontiers in Cellular and Infection Microbiology, 2019. **9**(251).DOI: http://doi.org/10.3389/fcimb.2019.00251.

12. Chiarelli, L.R., et al., *A multitarget approach to drug discovery inhibiting Mycobacterium tuberculosis PyrG and PanK.* Scientific Reports, 2018. **8**(1): p. 3187.DOI: http://doi.org/10.1038/s41598-018-21614-4.
13. Wlodarchak, N., et al., *In Silico Screen and Structural Analysis Identifies Bacterial Kinase Inhibitors which Act with β-Lactams To Inhibit Mycobacterial Growth.* Molecular Pharmaceutics, 2018. **15**(11): p. 5410-5426.DOI: http://doi.org/10.1021/acs.molpharmaceut.8b00905.
14. Kang, C.-M., et al., *The Mycobacterium tuberculosis serine/threonine kinases PknA and PknB: substrate identification and regulation of cell shape.* Genes & Development, 2005. **19**(14): p. 1692-1704.DOI: http://doi.org/10.1101/gad.1311105.
15. Ioerger, T.R., et al., *Identification of New Drug Targets and Resistance Mechanisms in Mycobacterium tuberculosis.* PLOS ONE, 2013. **8**(9): p. e75245.DOI: http://doi.org/10.1371/journal.pone.0075245.
16. Aggarwal, A., et al., *Development of a Novel Lead that Targets M. tuberculosis Polyketide Synthase 13.* Cell, 2017. **170**(2): p. 249-259.e25.DOI: http://doi.org/10.1016/j.cell.2017.06.025.
17. Liu, F., et al., *Structure-Based Optimization of Pyridoxal 5′-Phosphate-Dependent Transaminase Enzyme (BioA) Inhibitors that Target Biotin Biosynthesis in Mycobacterium tuberculosis.* Journal of Medicinal Chemistry, 2017. **60**(13): p. 5507-5520.DOI: http://doi.org/10.1021/acs.jmedchem.7b00189.
18. Jansen, R.S., et al., *Aspartate aminotransferase Rv3722c governs aspartate-dependent nitrogen metabolism in Mycobacterium tuberculosis.* Nature Communications, 2020. **11**(1): p. 1960.DOI: http://doi.org/10.1038/s41467-020-15876-8.
19. Cox, J.A.G., et al., *THPP target assignment reveals EchA6 as an essential fatty acid shuttle in mycobacteria.* Nature Microbiology, 2016. **1**(2): p. 15006.DOI: http://doi.org/10.1038/nmicrobiol.2015.6.
20. Willand, N., et al., *Synthetic EthR inhibitors boost antituberculous activity of ethionamide.* Nature Medicine, 2009. **15**(5): p. 537-544.DOI: http://doi.org/10.1038/nm.1950.
21. Munshi, T., et al., *Characterisation of ATP-Dependent Mur Ligases Involved in the Biogenesis of Cell Wall Peptidoglycan in Mycobacterium tuberculosis.* PLOS ONE, 2013. **8**(3): p. e60143.DOI: http://doi.org/10.1371/journal.pone.0060143.
22. VanderVen, B.C., et al., *Novel Inhibitors of Cholesterol Degradation in Mycobacterium tuberculosis Reveal How the Bacterium's Metabolism Is Constrained by the Intracellular Environment.* PLOS Pathogens, 2015. **11**(2): p. e1004679.DOI: http://doi.org/10.1371/journal.ppat.1004679.
23. Mori, G., et al., *Thiophenecarboxamide Derivatives Activated by EthA Kill Mycobacterium tuberculosis by Inhibiting the CTP Synthetase PyrG.* Chemistry & Biology, 2015. **22**(7): p. 917-927.DOI: http://doi.org/10.1016/j.chembiol.2015.05.016.
24. Baugh, L., et al., *Increasing the structural coverage of tuberculosis drug targets.* Tuberculosis, 2015. **95**(2): p. 142-148.DOI: http://doi.org/10.1016/j.tube.2014.12.003.
25. Connor, S.E., et al., *Structural and functional characterization of Mycobacterium tuberculosis triosephosphate isomerase.* Acta Crystallographica Section D, 2011. **67**(12): p. 1017-1022.DOI: http://doi.org/doi:10.1107/S0907444911042971.
26. Chaturvedi, S. and V. Bhakuni, *Unusual Structural, Functional, and Stability Properties of Serine Hydroxymethyltransferase from Mycobacterium tuberculosis.* Journal of Biological Chemistry, 2003. **278**(42): p. 40793-40805.DOI: http://doi.org/10.1074/jbc.M306192200.

27. Fivian-Hughes, A.S., J. Houghton, and E.O. Davis, *Mycobacterium tuberculosis thymidylate synthase gene thyX is essential and potentially bifunctional, while thyA deletion confers resistance to p-aminosalicylic acid.* Microbiology, 2012. **158**(2): p. 308-318.DOI: http://doi.org/10.1099/mic.0.053983-0.
28. Singh, V. and V. Mizrahi, *Identification and validation of novel drug targets in Mycobacterium tuberculosis.* Drug Discovery Today, 2017. **22**(3): p. 503-509.DOI: http://doi.org/10.1016/j.drudis.2016.09.010.
29. Fu, G., et al., *Crystal structure of DNA gyrase B′ domain sheds lights on the mechanism for T-segment navigation.* Nucleic Acids Research, 2009. **37**(17): p. 5908-5916.DOI: http://doi.org/10.1093/nar/gkp586.
30. Krieger, Inna V., et al., *Structure-Guided Discovery of Phenyl-diketo Acids as Potent Inhibitors of M. tuberculosis Malate Synthase.* Chemistry & Biology, 2012. **19**(12): p. 1556-1567.DOI: http://doi.org/10.1016/j.chembiol.2012.09.018.
31. Bruning, J.B., et al., *Structure of the Mycobacterium tuberculosis D-Alanine:D-Alanine Ligase, a Target of the Antituberculosis Drug D-Cycloserine.* Antimicrobial Agents and Chemotherapy, 2011. **55**(1): p. 291-301.DOI: http://doi.org/10.1128/aac.00558-10.
32. Sundaramurthi, J.C., et al., *TBDRUGS – Database of drugs for tuberculosis.* Tuberculosis, 2016. **100**: p. 69-71.DOI: http://doi.org/10.1016/j.tube.2016.06.006.
33. Goude, R., et al., *The Critical Role of embC in Mycobacterium tuberculosis.* Journal of Bacteriology, 2008. **190**(12): p. 4335-4341.DOI: http://doi.org/10.1128/jb.01825-07.
34. Huszár, S., et al., *N-Acetylglucosamine-1-Phosphate Transferase, WecA, as a Validated Drug Target in Mycobacterium tuberculosis.* Antimicrobial agents and chemotherapy, 2017. **61**(11): p. e01310-17.DOI: http://doi.org/10.1128/aac.01310-17.
35. Venugopala, K.N., et al., *Anti-Tubercular Properties of 4-Amino-5-(4-Fluoro-3- Phenoxyphenyl)-4H-1,2,4-Triazole-3-Thiol and Its Schiff Bases: Computational Input and Molecular Dynamics.* Antibiotics, 2020. **9**(9): p. 559.
